# Development of Novel Heparin/Protamine Nanoparticles Useful for Delivery of Exogenous Proteins In Vitro and In Vivo

**DOI:** 10.3390/nano10081584

**Published:** 2020-08-12

**Authors:** Shingo Nakamura, Naoko Ando, Masayuki Ishihara, Masahiro Sato

**Affiliations:** 1 Division of Biomedical Engineering, National Defense Medical College Research Institute, Saitama 359-8513, Japan; naoandokoro@gmail.com (N.A.); ishihara@ndmc.ac.jp (M.I.); 2 Section of Gene Expression Regulation, Frontier Science Research Center, Kagoshima University, Kagoshima 890-8544, Japan; masasato@m.kufm.kagoshima-u.ac.jp

**Keywords:** heparin/protamine particles, hepatocyte, intravenous injection, lacZ protein, nanoparticles, protein delivery, self-assembling

## Abstract

We previously reported that heparin/protamine particles (LHPPs) produced as nanoparticles through simple mixing of raw materials exhibit sustained protein release and can be retained in cells. In the present study, we modified LHPPs without employing any organic synthetic approach. The resulting LHPPs were re-named as improved LHPPs (*i*-LHPPs) and have the ability to retain cell-penetrating peptides (GRKKRRQRRRPPQ) based on electrostatic interactions. We examined whether *i*-LHPPs can introduce exogenous proteins (i.e., lacZ protein encoding bacterial β-galactosidase) into cultured cells in vitro, or into murine hepatocytes in vivo through intravenous injection to anesthetized mice. We found an accumulation of the transferred protein in both in vitro cultured cells and in vivo hepatocytes. To the best of our knowledge, reports of successful in vivo delivery to hepatocytes are rare. The *i*-LHPP-based protein delivery technique will be useful for in vivo functional genetic modification of mouse hepatocytes using Cas9 protein-mediated genome editing targeting specific genes, leading to the creation of hepatic disease animal models for research that aims to treat liver diseases.

## 1. Introduction

Introduction of genetic material is one of the representative techniques used to modify cell function. A foreign gene introduced into a cell can produce functional protein, via several processes, namely transcription (mRNA synthesis from the foreign gene), translation of mRNA into protein, and post-translational modifications. This cellular event is termed protein biosynthesis. In contrast, direct introduction of in vitro synthesized protein into a cell does not require protein biosynthesis. As a result, a protein introduced into a cell can rapidly exert its function. Furthermore, its function can be readily controlled by adjusting the protein dose [1]. However, in the case of protein delivery to a cell, maintaining the three-dimensional (3D) structure of the delivered protein itself is very important. If the 3D protein structure is destroyed and metamorphosed upon delivery into a cell, the protein will rapidly lose its function.

Generally, naked proteins introduced in vivo are rapidly degraded by proteolytic enzymes ubiquitously present in a given organism. Furthermore, naked proteins administered into the bloodstream may be difficult to be successfully delivered to target organs or tissues because they do not have specificity towards specific organs (or tissues) [1]. The use of a protein delivery carrier enabling protein encapsulation and subsequent release would overcome these limitations [2].

Small particles in the size range between 1 and 1000 nm, or sheets of that thickness, are generally designated as nanomaterials (or nanoparticles) and may be potentially harmful or beneficial to cells [3]. Generally, nanoparticles are potentially toxic to the human body; therefore, nanoparticles must undergo various removal reactions in vivo, depending on their size. For instance, nanomaterials smaller than 5.5 nm are subject to renal excretion [4]. Therefore, for in vivo applications, particularly when they are used as delivery carriers for intravenous administration, nanomaterials ranging between 100 and 200 nm would be desirable [3].

The liver is the largest organ in the body, and plays many roles in metabolism, excretion, detoxification, body fluid homeostasis, and digestion [5]. Therefore, hepatic diseases are highly likely to be fatal, and studies targeting the liver have been actively conducted [6]. In addition to basic research aimed at developing animal models of liver disease [7] and exploring liver disease treatments [8], the development of efficient methods to introduce foreign genes into mouse hepatocytes in vivo is important. Hydrodynamics-based gene delivery (HGD) is an efficient method that enables in vivo transfer of nucleic acids (NAs) into mouse hepatocytes via the caudal vein [9,10]. Unfortunately, few technical reports are available concerning protein introduction in vivo and in vitro [11,12,13]. For example, Rouet et al. demonstrated that protein introduction proceeds using protein delivery carriers capable of binding to the asialoglycoprotein receptor (ASGP-R) with a high degree of specificity [14]. ASGP-R is a hepatocyte-specific receptor, to which galactose specifically binds. Thus, such protein delivery carriers are modified to have galactose residues [15]. To create such protein delivery carriers requires technical skills and knowledge regarding organic synthesis, as exemplified by the introduction of modifying groups.

Here, we developed a novel biomaterial referred to as low-molecular-weight heparin/protamine particles (LHPPs) capable of interacting with NAs, proteins, and cells to protect them from proteolytic enzymes and thermal damage [16]. These materials are proven to exhibit sustained release of proteins [17,18,19] and are useful for retaining cells, which allows them to adapt to cell culture environments [20]. LHPPs can be synthesized by simply mixing two types of polymer chains (heparin and protamine) bearing opposite charges as monodisperse fine particles (also called micro- and nanoparticles). Their synthesis does not depend on organic reactions, and is performed via electrostatic interactions.

Since LHPPs can interact with proteins, we speculated that it might possible to deliver a target protein to hepatocytes in vivo if LHPPs are used as protein carriers. In this case, HGD, which is known as a technique allowing liver-directed gene introduction, can be used for targeted delivery into hepatocytes. Furthermore, we hypothesized that it might be possible to modify the surface of LHPPs through electrostatic interactions, because LHPPs have surface charge. For instance, it is possible to bind cell penetrating peptides (CPPs) [21], which can increase the efficiency of transmembrane transport by directly passing through a lipid membrane to the surface of LHPPs. This new type of LHPPs is referred to as improved LHPPs (*i*-LHPPs).

In this study, we examined whether *i*-LHPPs could be produced without organic synthesis, and the resulting *i*-LHPPs were used as protein carriers for delivery of a target protein to mouse hepatocytes via HGD.

## 2. Materials and Methods 

### 2.1. Preparation of LHPPs and i-LHPPs

LHPPs were prepared as previously described [17]. In brief, 14 mL of a low-molecular-weight heparin solution containing 10-fold diluted dalteparin (Fragmin: 6.4 mg/mL; Kissei Pharmaceutical Co., Tokyo, Japan) in phosphate-buffered saline without Ca^2+^and Mg^2+^, pH 7.2 [PBS(-)], was mixed with 6 mL of protamine solution containing 10-fold diluted protamine (10 mg/mL; Mochida Pharmaceutical Co., Tokyo, Japan) in PBS(-), and vortexed for 2 min. The final concentration of LHPPs was estimated to be 1.57 mg/mL [22]. The resulting LHPPs are referred to as “LHPPs 1.57 mg/mL” and can be stored at 4 °C for at least six months. For CPP, we used the human immunodeficiency virus-1 (HIV-1) viral protein Tat (trans-activator of transcription) (comprised of GRKKRRQRRRPPQ), which is known to have the ability to transport proteins into cells [21]. We hereinafter term this peptide ‘TAT’, and its synthesis was outsourced from Greiner Bio-One International (Tokyo, Japan). TAT was dissolved in PBS(-) at a concentration of 1 mg/mL and used as a stock solution. One milliliter of LHPPs 1.57 mg/mL was added with designated amounts of TAT, and the mixtures were kept on ice before measuring the particle size and zeta charge (electric charge on the nanoparticle surface) of the *i-*LHPPs using a particle size measurement system (ELSZ-1000; Otsuka Electronics Co. Ltd., Osaka, Japan). The resulting *i-*LHPPs containing TAT are referred to as “*i-*LHPPs 1.57 mg/mL” and can be stored at 4 °C for at least six months almost no change in charge or particle size. This experiment used the materials from three different batches, and a total of three experiments for each batch were performed.

### 2.2. Protein Delivery into Cultured Cells in Vitro

NIH3T3 cell [23] using in this study was obtained from ATCC (#CRL-1658) [24]. The cells were first seeded onto a well of a six-well gelatin-coated plate (#4810-020; Iwaki Glass Co., Tokyo, Japan) at a density of 10^6^ cells/well one day before introducing a protein and grown in 2 mL of Dulbecco ‘s modified Eagle’s medium (DMEM) (#11995065; Thermo Fisher Scientific K.K., Tokyo, Japan) supplemented with 10% fetal bovine serum (FBS) (Invitrogen Co., Carlsbad, CA, USA) at 37 °C in an atmosphere of 10% CO_2_.

Before introducing of *i*-LHPPs 1.57 mg/mL complexed with a protein, 1 µg of bacterial β-galactosidase (β-gal) (#072-04141; FUJIFILM Wako Pure Chemical Corp., Osaka, Japan) or RNase T1 (#109193; F. Hoffmann-La Roche Ltd., Basel, Switzerland) was added to 1 mL of *i*-LHPPs 1.57 mg/mL, and placed on ice for 30 min. Then, each mixture was added to the cell culture medium in a well. Notably, as reported in our previous study and in other studies, ~5 μg of protein can be immobilized on 1 mg of LHPPs [25,26,27]. One day after the addition of the *i*-LHPPs-protein mixture, cells were subjected to inspection for the presence of exogenously-added protein.

To detect the presence of β-gal in a cell, cells were subjected to cytochemical staining with X-gal, a substrate for β-gal, as described previously [28]. In brief, after washing with PBS(-), cells were fixed in 4% paraformaldehyde (PFA) in PBS(-) at 4 °C for 4 h, then, a solution of X-gal staining kit (GX10003; OZ Biosciences SAS, Marseille, France) was added and incubated at 30 °C for 24 h.

In the case of addition of RNase T1, cells were subjected to cell counting using a Cell Counting Kit (#343-07623; FUJIFILM Wako Pure Chemical Corp.), as described previously [20,29]. In brief, after washing with PBS(-), cells were incubated in 100 μL of fresh medium containing 10 μL of WST-1 reagent (containing tetrazolium salts) at 37 °C for 1 h. The optical density (OD) of each well was read at 450 nm using a microplate spectrophotometer (Multiskan FC; Thermo Fisher Scientific K.K.).

### 2.3. Protein Delivery into Murine Hepatocytes via HGD

This experiment involved HGD-based in vivo delivery of proteins using mice. Institute of Cancer Research (ICR) male mice (7-weeks old) were purchased from Japan SLC Inc. (Hamamatsu, Japan), maintained on a 12 h light/dark schedule (lights on from 07:00 to 19:00), and were allowed food and water ad libitum. All animal experiments were performed at the National Defense Medical College (Saitama, Japan) in accordance with the guidelines of the *National Defense Medical College Committee*, and approved by the *Care and Use of Laboratory Animals* (permission no. 17064; valid from 27 July 2017 to 31 March, 2020). All efforts were made to reduce the number of animals used and to minimize their suffering (n = 3 in each group).

First, 1 mL of a stock solution containing 10 µg of β-gal and 100 µg of TAT in *i*-LHPPs 1.57 mg/mL was prepared. Prior to injection, mice were subjected to sufficient anesthesia by intraperitoneal (IP) injection of three combined anesthetics [medetomidine (0.75 mg/kg; Nippon Zenyaku Kogyo Co. Ltd., Fukushima, Japan), midazolam (4 mg/kg; Sandoz K.K., Tokyo, Japan), and butorphanol (5 mg/kg; Meiji Seika Pharma Co., Ltd., Tokyo, Japan)]. HGD was performed according to methods described previously [30,31,32]. Briefly, anesthetized mice were intravenously injected with PBS(-) containing 10 µL of stock solution using a syringe (3 mL Luer lock type; Nipro, Inc., Osaka, Japan) fitted with a 30-gauge needle (Dentronics Co., Ltd., Tokyo, Japan). The volume (mL) used for HGD is calculated as one-tenth of the body weight (g). For example, for 30 g of a mouse it requires 3 mL of *i*-LHPPs-containing solution. Injections were performed at a constant injection speed via the tail vein and completed within 10 s by the same researcher in order to avoid artefactual effects in each experiment. After intravenous injection, anesthetized mice were recovered by subcutaneous injection of atipamezole (3.75 mg/kg; Nippon Zenyaku Kogyo Co. Ltd.), a medetomidine antagonist, and then maintained on an electric plate warmer for recovery from anesthesia.

Two days after injection, the mice were euthanized and subjected to perfusion fixation, according to Nakamura et al. [33]. Liver samples were dissected out and fixed in 4% PFA in PBS(-) at 4 °C for one day and then subjected to standard immunohistochemical staining using rabbit anti-β-gal antibody with the aid of a professional company (KYODO BYORI. Inc., Kobe, Japan). In brief, cryostat specimens were incubated with rabbit anti-β-gal polyclonal antibody (diluted 1/200; #100-4136; Rockland Immunochemicals, Inc., Limerick, PA, USA) (as the primary antibody) at 4 °C for one day, and subsequently with peroxidase-labeled anti-rabbit IgG polyclonal antibody (#424144; NICHIREI BIOSCIENCE INC, Tokyo, Japan) (as the secondary antibody) at 4 °C for 3 h. The specimens were then color-developed using 3,3′-diamino benzidine (DAB) solution. After counterstaining with 4′,6-diamidino-2-phenylindole (DAPI), the immunostained specimens were inspected using a phase-contrast microscope (#BZ-8000; Keyence Co., Osaka, Japan).

### 2.4. Statistical Analysis

The zeta potential [the electric charge that develops at the interface between a solid surface (nanoparticle) and its liquid medium], particle size, and number of cells are presented as means ± standard deviation. Statistical analysis was performed using unpaired Student’s *t*-test, and one-way factorial analysis of variance. Scheffe’s post-hoc test was used for multiple comparisons. The *p*-values were calculated using JMP14 for Windows software (SAS Institute Japan Ltd., Tokyo, Japan). A *p*-value of <0.05 was considered to indicate statistical significance. Values of *p* < 0.01 are marked with a double asterisk; <0.05 with a single asterisk.

## 3. Results

### 3.1. Preparation of i-LHPPs

**Figure 1:**
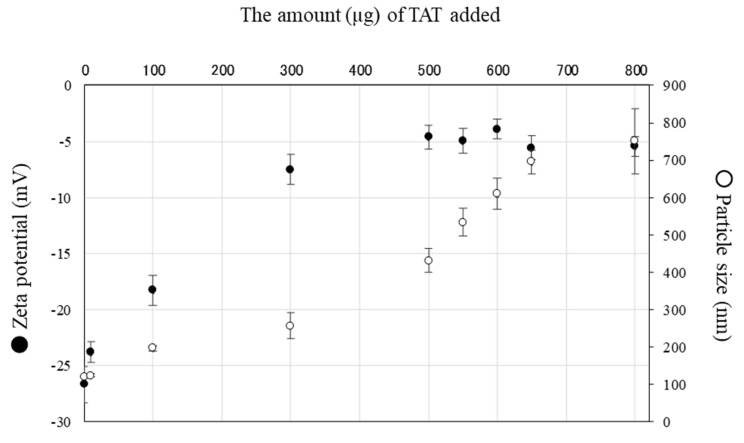
Relationship between LHPPs particle size and zeta potential after addition of TAT as CPP. Black circles in the graph indicate zeta potential (mV) (shown at left vertical axis); white circles indicate particle size (nm) (shown at the right vertical axis). The horizontal axis indicates the amounts (μg) of TAT added. As TAT (up to 300 μg) was added to LHPPs at 1.57 mg/mL, both zeta potential and particle size increased. However, although the zeta potential error remained unaltered, the addition of >300 μg of TAT caused increased particle size error, suggesting particle instability due to size heterogeneity.

We added TAT (comprised of GRKKRRQRRRPPQ) to LHPPs to create *i*-LHPPs. Since the TAT protein used contains many arginine residues, the peptide itself is thought to be cationic. Thus, it is likely that the addition of TAT to LHPPs will result in a charge shift towards the cationic state. Measurement of the zeta potential in the TAT-containing LHPPs demonstrated that as the dosage of TAT increased, the surface charge of LHPPs shifted from negative (approximately between −25 mV and −30 mV) to the neutral charge side, stabilizing at −5 mV (Figure 1). The particle size remained stable, although a slight fluctuation in the particle size (120.27 ± 4.46 nm, 123.47 ± 4.41 nm, and 196.7 ± 6.75 nm) was observed during the addition of 0, 10, and 100 µg of TAT, respectively. However, the error range of particle size (256.17 ± 33.89 nm to 751.13 ± 86.94 nm) increased, along with an increase in the amount of TAT from 300 to 800 µg. These findings suggested an interaction between TAT and LHPPs. Furthermore, particle stability is guaranteed when 100 µg of TAT is added to a solution containing 1.57 mg/mL of LHHPs. Thus, hereinafter we decided to use *i*-LHPPs at a ratio of 100 µg of TAT to 1.57 mg/mL of LHHPs.

### 3.2. Incorporation of Proteins Using i-LHPPs into NIH 3T3 Cells

**Figure 2:**
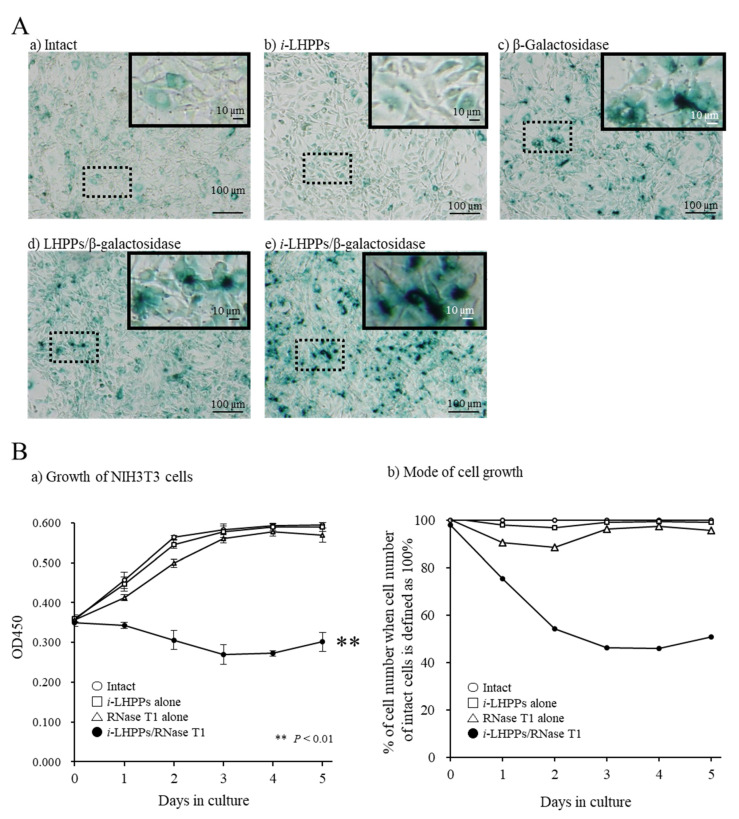
*i*-LHPPs-mediated protein delivery in vitro. (**A**) NIH3T3 cells stained in the presence of x-gal for β-galactosidase (β-gal) activity after in vitro protein delivery. In each microphotograph, the upper right image represents the magnified image of the dotted square area. Notably, in untreated (intact) cells (**A-a**), or cells treated with *i*-LHPPs alone (**A-b**), nonspecific positive reactions were observed, probably due to endogenous β-gal activity. A slight increase in blue deposition was noted in cells treated with β-gal alone (**A-c**), or in those treated with LHPPs/β-gal complexes (**A-d**). However, cells exposed to *i*-LHPPs/β-gal complexes exhibited distinct blue cytoplasmic deposits (**A-e**). (**B**) Survivability of NIH3T3 cells after in vitro protein (RNase T1) delivery. (**B-a**) Cell growth in groups of intact cells (shown by white circles), cells treated with *i*-LHPPs alone (shown by squares), cells treated with RNase T1 alone (shown by triangles), or cells treated with *i*-LHPPs/RNase T1 complex (black circles). Cell growth was assessed by measuring optical absorbance at 450 nm, and represented by OD values on the longitudinal axis. Data are shown as means ± SD. ** indicates *p <* 0.01 when OD 450 nm values were evaluated between cells treated with *i*-LHPPs/RNase T1 complex and those treated with none (intact), *i*-LHPPs alone, or RNase T1 only. (**B-b**) Cell survivability for each treatment group when the survival rate of cells in the intact group on each culture day was defined as 100%. Notably, the number of cells after treatment with *i*-LHPPs/RNase T1 complexes (black circle) was greatly reduced after up to three days in culture, suggesting that exogenous protein is indeed incorporated by cells when delivered in the form of *i*-LHPPs/protein complexes. Measurements were repeated three times on different days.

To test the possibility that *i*-LHPPs enabled the incorporation of a protein into cultured cells in vitro, NIH3T3 cells were exposed to *i*-LHPPs coupled with β-gal for 24 h at 37 °C. After that, the cells were fixed and then subjected to cytochemical staining in the presence of x-gal, a β-gal substrate. As a result, numerous cells exhibited blue cytoplasmic deposits when the *i*-LHPPs/β-gal complex was employed (Figure 2A-e). Notably, in the groups where β-gal alone was used, or when the LHPPs/β-gal complexes (without TAT) were used, x-gal-positive cells were observed (Figure 2A-c,d). However, the rate of x-gal-positive cells in the latter two groups was significantly lower than that in the *i*-LHPPs/β-gal complex-treated groups (c,d, vs. e in Figure 2A). Only marginal staining was seen in cells treated with *i*-LHPPs alone (Figure 2A-b), and in those without any treatment (intact) (Figure 2A-a), suggesting residual endogenous β-gal activity present in NIH3T3 cells.

Next, to confirm that exogenous protein delivered via *i*-LHPPs is indeed internalized inside cells, RNase T1 was used in this *i*-LHPPs-based protein delivery system. RNase T1 is known to elicit cell death caused by inhibition of RNA synthesis when delivered inside a cell [34]. Incubation of NIH3T3 cells in the presence of the *i*-LHPPs/RNase T1 complex for up to five days at 37 °C resulted in retardation of cell growth, which can be assessed by staining cells with tetrazolium salt-containing solution (black circles in Figure 2B-a). This mode of cell growth in the group treated with the *i*-LHPPs/RNase T1 complex was significantly different from that in the other groups (treatment with RNase T1 alone, LHPPs/RNase T1 complex, or none) (Figure 2B-a). If the survival rate of cells in the intact group at each day of culture was defined as 100%, cells treated with the *i*-LHPPs/RNase T1 complex reached approximately 50% survival after 3 days in culture (Figure 2B-b). Notably, cells treated with RNase T1 alone exhibited transient reductions in cell numbers (Figure 2B-b). These findings clearly suggested that exogenous protein is incorporated by a cell when delivered in the form of an *i*-LHPPs/protein complex.

### 3.3. Introduction of Protein to Mouse Hepatocytes via the Tail Vein Using i-LHPPs

**Figure 3:**
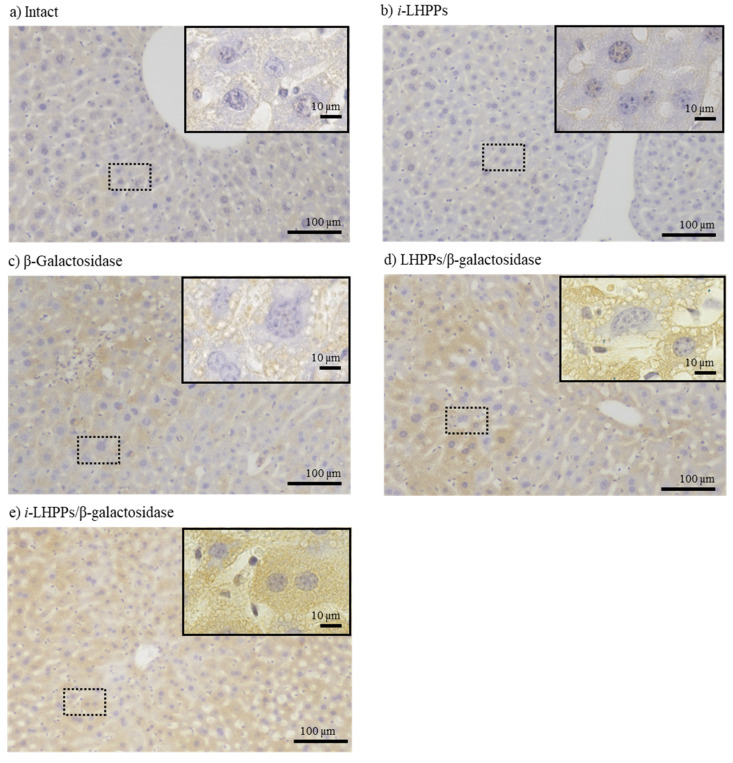
Immunocytochemical staining of mouse liver two days after HGD-based protein (β-galactosidase, β-gal) delivery in vivo. After staining with antibodies, specimens were subjected to 3,3′-diamino benzidine (DAB)-based coloration reactions, which cause generation of brown deposits if the specimens have cytoplasmic β-gal. Blue deposits indicate cell nuclei revealed by 4′,6-diamidino-2-phenylindole (DAPI) staining. In each microphotograph, the upper right image represents the magnified image of the dotted square area. Extensive and substantial brown deposits are discernible when *i*-LHPPs/β-gal complexes were used for HGD (**e**). In contrast, in specimens where no protein was introduced (**a**), or in those where *i*-LHPPs alone were introduced (**b**), poor coloration of immunoreactive substances was observed (which may reflect endogenous nonspecific reactivity against the antibody). Introduction of β-gal alone (**c**) or LHPPs/β-gal complexes (**d**) resulted in generation of minor amounts of immunoreactive substances in hepatocytes.

To examine whether *i-*LHPP*-*based protein delivery is also possible in vivo, we introduced the *i*-LHPPs/β-gal complex intravenously using the HGD method, which is known to be effective for targeted delivery of NAs to hepatocytes [9,10]. Two days after protein delivery, HGD-treated mice were subjected to perfusion fixation, liver dissection, and subsequent post-fixation prior to histochemical staining for the presence of the introduced β-gal using anti-β-gal antibody. If β-gal is successfully delivered to hepatocytes and taken up, liver specimens should exhibit brown cytoplasmic deposits [products generated after enzymatic reaction involving DAB and peroxidase (to which the secondary antibody had been conjugated)]. Consequently, extensive and substantial brown deposits were recognizable throughout the liver specimens obtained after delivery of *i*-LHPPs/β-gal complexes (Figure 3e). Minor deposition of β-gal-reacted products was also observed in the specimens derived from delivery of β-gal alone (Figure 3c), or in those from LHPPs/β-gal complexes (without using TAT) (Figure 3d), whereas the extent was less than that seen in specimens derived from delivery of the *i*-LHPPs/β-gal complexes (Figure 3c,d vs. Figure 3e). No such deposits were noted in untreated (intact) specimens (Figure 3a), or in those that had been given *i*-LHPPs alone (Figure 3b). From these findings, it was found that it is possible to deliver proteins into hepatocytes without loss of integrity when *i*-LHPPs are used as protein carriers.

## 4. Discussion

LHPPs are unique materials that are developed and produced using our methodology, which is based on the simple and clear hypothesis that mixing a polymer with positive charge and that with negative charge might form complexes via electrostatic interactions. In our previous study, we reported the usefulness of LHPPs complexed with growth-stimulating proteins to enhance the biological function of certain specific cells through interaction between the cells and LHPPs. For example, we reported that local administration of LHPPs carrying recombinant fibroblast growth factor-2 (FGF-2), known as an angiogenic factor, resulted in the controlled release of FGF-2, finally causing angiogenesis at the injection site of a mouse [17,18]. In this case, it was impossible to introduce a protein of interest into a cell. If this process was successful, the utility of LHPPs with other proteins could be greatly expanded. In this study, we realized the aforementioned idea by developing a new protein carrier (*i*-LHPP), which was easily produced by mixing CPP with LHPPs. This is a novel achievement that is different from the results of our previous work. The resulting complexes are generally referred to as “polyelectrolyte complexes (PECs)” [16,35,36], with both positive and negative surface charges. Accordingly, LHPPs appear to have similar surface conditions. Analysis of the properties of LHPPs using a particle size measurement system suggested that the surface charge of LHPPs remained stable, with a charge of approximately –30 mV (see Figure 1), which indicates that LHPPs are predominantly occupied by negative charges. This finding, in turn, prompted us to suppose that a peptide with net positive charge could be easily introduced into LHPPs through electrostatic interactions with modification groups. In the present study, we decided to use TAT as a modification group of CPPs [21] in order to enhance the cell permeability of LHPPs. When we added TAT (100 µg) to LHPPs (1.57 mg/mL), the zeta potential value was found to change from negative to neutral (Figure 1). This phenomenon suggested molecular interactions involving TATs and LHPPs.

To test whether the resulting LHPPs mixed with TAT (defined as *i*-LHPPs) have the ability to transport a protein into a cell, NIH3T3 cells were co-incubated with medium containing *i*-LHPPs/β-gal complex for 24 h. As expected, blue deposits were more frequently discernible on the cells treated with *i*-LHPPs/β-gal complexes than on those treated with β-gal alone, or with LHPPs/β-gal complexes (see Figure 2A). It may be claimed that the extensive accumulation of blue deposits on cells treated with *i-*LHPPs/β-gal complexes could be ascribed to simple attachment of these complexes on the surface of NIH3T3 cells, but not to the internalization of β-gal inside a cell. To eliminate this possibility, we next introduced *i*-LHPPs/RNase T1 complexes into NIH3T3 cells. It is well known that intracellular uptake of RNase T1, which interferes with cellular RNA synthesis, causes apoptosis-based cell death [34]. Incubation of NIH3T3 cells in a medium containing *i*-LHPPs/RNase T1 complexes led to reduced cell survival, as shown in Figure 2B. In contrast, treatment of cells with other reagents (*i*-LHPPs, RNase T1 alone, or LHPPs/RNase T1 complexes) did not affect NIH3T3 cell survival (Figure 2B). From these results, it can be concluded that *i*-LHPPs enable the internalization of a target protein inside a cell. Similar to other reagents used with CPP for protein delivery [37], the mode of protein internalization mediated by *i*-LHPPs may be as follows: (1) attachment of protein (β-gal, or RNase T1 in this study) to the cell surface, and (2) internalization of the protein by endocytosis (micropinocytosis), a general process by which cells absorb external material by engulfing it within the cell membrane [38]. Since the size of *i*-LHPPs used in this study was 100–200 nm (see Figure 1), it is reasonable to consider that these molecules can be internalized via micropinocytosis, namely via vesicle-mediated endocytosis, as shown in the case of another CPP fusion protein. The *i*-LHPPs that are uptaken by a cell through micropinocytosis will then be degraded within intracellular lysosomes, as suggested by Commisso et al. [39].

Our *i*-LHPPs are useful for targeted protein delivery to hepatocytes in vivo, and were also confirmed to be effective using HGD, a well-known technology enabling efficient gene delivery and targeting mouse hepatocytes [9,10]. HGD is performed by intravenous injection of a large amount of solution at once to rapidly increase intravenous pressure to pierce the pores (approximately 100 nm in mice) of hepatocytes and introduce NAs administered into the blood into hepatocytes [40]. Since the particle sizes of LHPPs and *i*-LHPPs are expected to be > 100 nm, it is unlikely that these materials are directly introduced into hepatocytes by passing through cell pores. The probability of this event is that *i*-LHPPs/protein (β-gal) complexes provided by HGD accumulates in the murine liver and adheres to the pore surface of hepatocytes by the action of TAT as CPP, and is then taken up by hepatocytes through the mechanism of cellular phagocytosis. Notably, successful HGD-based delivery of NAs into mouse hepatocytes has been identified by many laboratories; however, to our knowledge, reports of the delivery of proteins by the HGD technique appear to be few, probably due to instability in the blood and poor membrane permeability [1]. In this context, the *i*-LHPPs/HGD-based protein delivery method is important for delivering polypeptides of interest to the liver in vivo. Furthermore, this technique will provide a novel approach to create animal disease models, or to develop techniques to cure diseases in such models. It may also be possible to apply this technology to the field of in vivo genome editing, which has been extensively used in recent years for research and disease treatment. For instance, tail vein-mediated injection of *i*-LHPPs coupled with Cas9 protein (now commercially available) and single guide RNA, which is a synthetic RNA comprised of CRISPR RNA (crRNA) and trans-activating crRNA (tracrRNA) and can be easily obtained by outside sourcing, will be possible to realize this idea.

The most remarkable advantage of using *i*-LHPPs is that it does not require any organic synthetic process, which is laborious and time-consuming. *i*-LHPPs are produced through “simple mixing of materials”, which makes it easier to perform in vivo protein transfer experiments. In this study, we used TAT as CPP to enhance the incorporation rate of a target protein into cells. Unfortunately, CPP does not exhibit affinity for any specific cells or tissues. In this case, instead of CPP, the use of a tissue-specific protein, as exemplified by ASGP-R [14], appears to be of interest. LHPPs incorporating ASGP-R would show preferential affinity for the liver when they are provided intravenously. We believe that HGD-based protein delivery using LHPPs coupled with ASGP-R could be more effective than HGD-based protein delivery using *i*-LHPPs. Notably, organ- or cancer cell-specific receptors exist [10]. If we use such receptors as guide proteins for LHPPs, it might be possible to produce *i*-LHPPs with tissue or organ specificity. Alternatively, the use of mannose as a modifying group that can bind specifically to mannose receptors expressed in the lung [41] would be of interest. In this case, *i*-LHPPs would be used as “inhaled drug delivery material”, which does not require bloodstream-mediated introduction of drugs such as HGD.

We used RNase T1 (MW = 11 kDa) and β-gal (MW = 54 kDa) for protein delivery with our *i*-LHPPs. In our previous study, we had succeeded in the controlled release of recombinant FGF-2 (MW = 17 kDa) from the LHPPs/FGF-2 complex that was injected in vivo [17,18]. We believe that it is possible to deliver up to 5 μg of protein per 1 mg of LHPPs with a one-shot injection [25,26,27]. This appears to be solely due to the nature of LHPPs, which mainly comprise heparin and easily interact with cationic proteins, as exemplified by heparin-binding protein. Notably, since the surface of LHPPs has positive and negative charges, it is highly likely that LHPPs can potentially retain anionic proteins. Thus, it is conceivable that *i*-LHPPs have a similar property to that of LHPPs. In this context, it would be worthwhile to examine the types of proteins trapped by *i*-LHPPs, in view of increasing their utility.

HGD is widely accepted as an efficient method that enables the in vivo transfer of NAs into mouse hepatocytes via the caudal vein [42,43]. Gene delivery to the kidneys and lungs is also possible with this method, although the transfection efficiency is low and the number of transferred NAs appears to be low, in the case of a successful transfection [9]. Similarly, it is reasonable to consider that protein delivery to these organs can be successfully achieved by using *i*-LHPPs. Unfortunately, we failed to detect the presence of the introduced protein (β-gal) in both the kidneys and the lungs (unpublished data). This failure appears to be closely associated with the presence of TAT as CCP included in *i*-LHPPs, because it is known that the composition and organization of the extracellular matrix can affect the tissue localization pattern of substances that are introduced in vivo [44,45].

Notably, HGD itself appears to be infeasible in humans because it requires the rapid administration of a large amount of solution via the tail vein. Suda and Liu [46] reported that fluid overload in systemic circulation induces irregular cardiac function, leading to transient heart failure. However, the application of this technology to large animals is also being attempted. For instance, the feasibility of local administration with the aid of a catheter, instead of systemic administration, is now considered a safer approach to achieve gene delivery to individuals [47]. This way, catheter-based local administration of *i*-LHPPs in large animals would be valuable, in view of increasing the utility of *i*-LHPPs.

Determination of the optimal dose of *i*-LHPPs is important for safe in vivo administration. Since LHPPs mainly comprise heparin, there is a concern regarding occasional bleeding when these substances are intravenously administered to mice. We already checked this possibility and found almost no sign of occasional bleeding after administration of LHPPs [17]. Furthermore, we did not observe any bleeding in the liver specimens that are thought to be mostly affected by the HGD treatment (Figure 3,b,d,e). Although there is a still room to take steps to avoid possible bleeding elicit by LHPPs, we believe that as long as *i*-LHPPs (which are constructed at a heparin/protamine ratio determined in this study) are used, blood leakage is not possible.

Regarding possible changes in body weight and behavior during the experimental period of two days after the administration of one shot of *i*-LHPPs, we did not observe any noticeable changes in the mice. However, repeated administration of *i*-LHPPs may elicit unwanted immunological issues. In this context, we plan to evaluate this possibility through the periodic collection of mouse serum.

## 5. Conclusions

We developed a novel protein carrier system, termed *i*-LHPPs, allowing delivery of a target protein into cells both in vitro and in vivo. This system can be distinguished from other existing systems based on organic synthesis for creating biomaterials, which always require skilled techniques and advanced expertise. *i*-LHPPs can be created via an extremely easy method: it is sufficient to mix raw materials in two steps; mixing protamine and low-molecular-weight heparin to produce LHPPs in a first step, and mixing LHPPs and CPP in a second step. With HGD-based delivery of *i*-LHPPs, we introduced a protein (β-gal) into mouse hepatocytes in vivo. By replacing CPP with other positively charged short peptides, such as receptor proteins in *i*-LHPPs, it would be possible to deliver a target protein into tissues or organs other than the liver. Thus, our protein carrier system, which does not rely on organic synthesis, has great potential for use in various research fields, including animal biotechnology and biomedicine. On the other hand, there are some subjects to be overcome in future, which include physicochemical characterization of *i*-LHPPs, as exemplified by protein loading efficiency and stability in vitro and in vivo. These data will be especially important when *i*-LHPPs produced by a simple synthetic method are applied to various research fields.
